# Case Report: Effect of setmelanotide treatment in a young patient with acquired hypothalamic obesity following *Escherichia coli* sepsis and meningoencephalitis with brain abscess

**DOI:** 10.3389/fendo.2026.1796227

**Published:** 2026-06-08

**Authors:** Hajar Dauleh, Khalid Hussain

**Affiliations:** 1 Division of Endocrinology, Department of Pediatric Medicine, Sidra Medicine, Doha, Qatar; 2 Department of Endocrinology, Weill Cornell Medicine-Qatar, Doha, Qatar

**Keywords:** acquired hypothalamic obesity, childhood obesity, MC4R agonist, meningoencephalitis, setmelanotide

## Abstract

Acquired hypothalamic obesity (aHO) is a rare and severe condition caused by injury to hypothalamic centers regulating appetite, energy balance, and neuroendocrine function. It is characterized by rapid weight gain, hyperphagia, and significant metabolic complications, and current treatment options remain limited. We report the case of a 22-month-old child who developed severe acquired hypothalamic obesity following neonatal *Escherichia coli* sepsis complicated by meningoencephalitis and brain abscess. Treatment with the melanocortin-4 receptor agonist setmelanotide was initiated at 0.25 mg daily and gradually titrated to 1.5 mg. Over 13 months of treatment, the patient experienced stabilization and a subsequent reduction in body weight, improvement in liver enzymes and lipid profile, increased motor activity, and marked improvements in interaction and quality of life. The most pronounced clinical response occurred at doses ≥1.25 mg daily. This case represents, to our knowledge, the youngest reported patient treated with setmelanotide and the first case of inflammation-related acquired hypothalamic obesity treated with this therapy. These findings suggest that setmelanotide may represent a promising therapeutic strategy for selected patients with acquired hypothalamic obesity beyond currently approved genetic indications.

## Introduction

Hypothalamic obesity (HO) is a disorder characterized by hyperphagia, rapid weight gain, and metabolic complications resulting from injury to hypothalamic centers regulating energy homeostasis, autonomic function, and neuroendocrine signaling (1, 2). HO can be broadly classified into genetic and acquired forms. Genetic HO results from defects affecting components of the leptin–melanocortin signaling pathway, whereas acquired hypothalamic obesity (aHO) develops following structural or inflammatory injury to hypothalamic regions involved in energy balance (2). Causes of aHO include suprasellar tumors and their treatment, traumatic or hypoxic–ischemic brain injury, infection, inflammation, and other acquired insults. Regardless of etiology, HO is frequently resistant to conventional therapeutic approaches.

The leptin–melanocortin pathway plays a central role in appetite regulation and body weight control. Leptin, secreted by adipocytes in proportion to fat mass, acts on hypothalamic neurons by binding to the leptin receptor (LEPR) on pro-opiomelanocortin (POMC) and agouti-related peptide (AgRP) neurons in the arcuate nucleus. Activation of LEPR on POMC neurons promotes production of α-melanocyte-stimulating hormone (α-MSH), which stimulates melanocortin-4 receptor (MC4R)–expressing neurons to induce satiety and reduce food intake. In parallel, leptin suppresses AgRP neurons, which otherwise inhibit MC4R signaling and stimulate appetite. Disruption of this pathway results in impaired satiety signaling and severe obesity. Genetic defects involving LEPR, POMC, PCSK1, or MC4R highlight the importance of melanocortin signaling in pediatric energy balance.

Although hypothalamic injury in aHO disrupts multiple neural circuits involved in energy homeostasis, residual melanocortin signaling pathways may remain partially functional. This provides a mechanistic rationale for targeting MC4R signaling as a therapeutic strategy in selected patients with acquired hypothalamic obesity, as pharmacologic activation of MC4R may partially bypass upstream hypothalamic injury and restore downstream satiety signaling.

Setmelanotide, is approved for patients aged ≥2 years with rare genetic disorders affecting the melanocortin pathway, including POMC, PCSK1, and LEPR deficiencies, as well as Bardet–Biedl syndrome (3). By mimicking α-MSH, setmelanotide activates MC4R signaling and promotes appetite regulation and energy expenditure. Although hypothalamic injury may affect MC4R-expressing regions in aHO, emerging evidence suggests that activation of the melanocortin pathway may provide therapeutic benefit in selected patients.

In a phase 2, open-label, multicentre trial, Roth et al. evaluated setmelanotide in 18 adolescents and adults aged 12–40 years with acquired hypothalamic obesity, predominantly secondary to craniopharyngioma or other suprasellar tumors. Treatment resulted in a mean reduction in body mass index of approximately 15.8% at 16 weeks and a decrease in hunger scores of 2.4 points on a standardized hunger scale (4). These findings were supported by a randomized, double-blind, placebo-controlled phase 3 trial evaluating setmelanotide in patients aged 6–40 years with tumor- or treatment-related acquired hypothalamic obesity, which demonstrated improvements in body mass index and hyperphagia-related outcomes compared with placebo (5).

More recently, the VENTURE phase 3 trial evaluated setmelanotide in children aged 2–5 years with rare MC4R pathway–associated genetic obesity, demonstrating favourable safety and efficacy over one year of treatment (6). However, the use of setmelanotide in children younger than 2 years with acquired hypothalamic obesity has not been reported, particularly in cases related to inflammatory brain injury.

Here, we report a case of severe acquired hypothalamic obesity secondary to neonatal *Escherichia coli* meningoencephalitis with brain abscess successfully treated with setmelanotide for 13 months in a 22-month-old child.

## Case presentation

We report a 22-month-old male child, born at term by vaginal delivery to consanguineous parents (first cousins), with a birth weight of 2700 g. He is the fifth of six siblings and had an unremarkable perinatal course. At six days of age, he developed fulminant *Escherichia coli* sepsis complicated by meningoencephalitis and a brain abscess, necessitating a prolonged neonatal intensive care unit stay of approximately four months. The meningoencephalitis subsequently resolved; however, it resulted in significant structural brain injury involving hypothalamic regions, as well as central adrenal insufficiency and arginine vasopressin deficiency (AVP-D). The patient subsequently developed rapid early-onset weight gain consistent with acquired hypothalamic obesity, which became clinically evident during infancy.

The patient’s follow-up period spanned two different anthropometric frameworks. In children younger than 2 years, weight-for-length is the recommended index for assessing adiposity, whereas in children aged ≥2 years, BMI-for-age becomes the appropriate measure. As these indices are derived from different growth standards and their z-scores are not directly interchangeable, a single continuous BMI z-score trajectory was not presented.

Excessive weight gain was noted as early as seven weeks of age. At three months, his weight was 6.3 kg (SD −0.76), which rapidly increased to 23.8 kg at 12 months (SD + 8.42), 32.5 kg at 18 months (SD + 9.56), and 35.8 kg at 22 months of age (SD + 9.35). This was accompanied by marked hyperphagia with persistent crying and constant demand for feeding.

On physical examination at 22 months post birth age, the patient was vitally stable and exhibited severe obesity without identifiable dysmorphic features. Neurologically, he demonstrated poor interaction, minimal visual tracking, and the absence of babbling. He had poor head control and difficulty bringing his hands to the midline, largely attributable to pronounced truncal adiposity. Spontaneous movements were minimal. His hands were predominantly fisted, although he was able to open them, and he did not reach for or grasp objects. No spontaneous lower limb movements were observed, and there were no contractures or signs of hyperreflexia. Cardiovascular examination revealed normal first and second heart sounds. The abdomen was soft, and genital examination showed normal male genitalia. No dysmorphic cutaneous stigmata were noted. However, severe friction within skin folds due to marked adiposity resulted in erythema and superficial erosions.

Laboratory investigations at 22 months of age demonstrated markedly elevated liver enzymes, with alanine aminotransferase (ALT) of 330 IU/L (reference range: 10–25 IU/L) and aspartate aminotransferase (AST) of 228 IU/L (reference range: 23–46 IU/L). Significant dyslipidemia was also present, with total cholesterol of 7.8 mmol/L, Low-Density Lipoprotein Cholesterol (LDL) cholesterol of 5.6 mmol/L, non-High-Density Lipoprotein Cholesterol (non-HDL) cholesterol of 5.7 mmol/L, and triglycerides of 3.0 mmol/L, while HDL cholesterol remained within the normal range at 1.5 mmol/L. Serum leptin was markedly elevated at 80 ng/mL. Abdominal ultrasonography demonstrated hepatomegaly with increased hepatic echogenicity and moderate fatty infiltration, findings consistent with metabolic dysfunction–associated steatotic liver disease (MASLD). Brain magnetic resonance imaging revealed extensive cystic encephalomalacia with deep grey matter gliosis.

**Figure 1:**
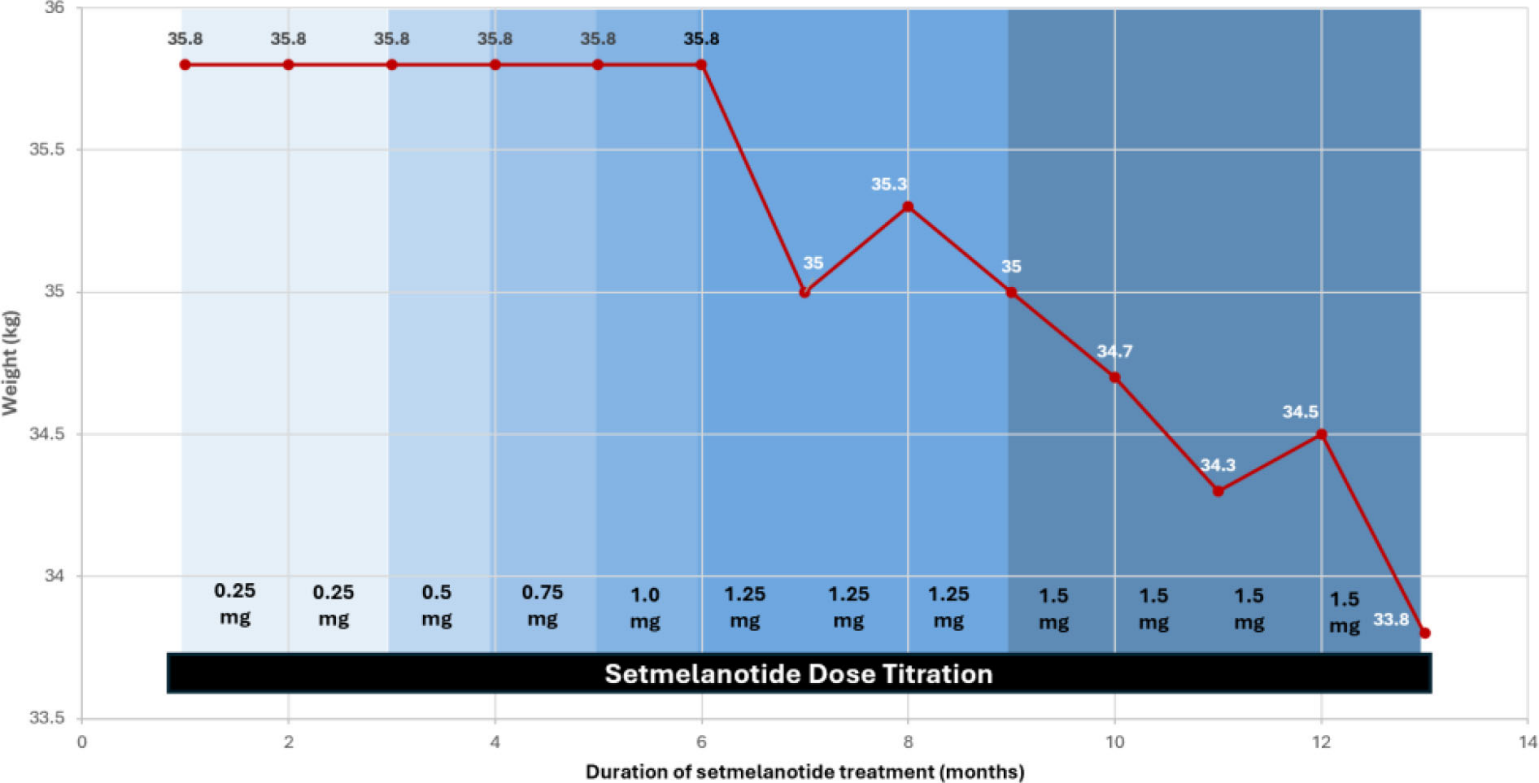
Weight trajectory during 13 months of setmelanotide therapy with dose titration in a child with acquired hypothalamic obesity. Treatment was initiated at 0.25 mg daily and gradually escalated to 0.5 mg, 0.75 mg, 1.0 mg, 1.25 mg, and 1.5 mg according to clinical response and tolerability.

Setmelanotide was initiated at 22 months of age using a standardized dose initiation and titration protocol (). Dose escalation was guided by clinical response and tolerability, with response defined as a weight reduction of approximately 0.5–1.5 kg over 4 weeks. Treatment was initiated at 0.25 mg once daily by subcutaneous injection for the first two months. The dose was then escalated sequentially to 0.5 mg for one month, 0.75 mg for one month, and 1.0 mg for one month. Further titration increased the dose to 1.25 mg daily, which was maintained for three months, followed by escalation to 1.5 mg daily for the remainder of the treatment period (**Figure 1**). Body weight initially plateaued at 35.8 kg during the early treatment phase. After approximately 7 months of therapy, while receiving 1.25 mg daily, body weight began to decrease, reaching 35.0 kg. Significant improvements in motor activity, interaction with caregivers, vocalization, and overall developmental engagement were also observed.

Quality of life was assessed using the Pediatric Quality of Life Inventory (PedsQL). After 9 months of treatment, substantial improvements were observed across multiple domains. Scores decreased from 51 to 22 for physical functioning, from 36 to 8 for emotional functioning, and from 20 to 8 for social functioning, indicating improved functional status and reduced symptom burden. Family impact scores improved from 86 to 28, reflecting reduced caregiving burden and improved overall family functioning.

Treatment was associated with marked improvement in metabolic parameters. ALT decreased from 330 IU/L at baseline to 221 IU/L at 6 months and normalized to 15 IU/L at 12 months (reference range 10–25 IU/L). AST decreased from 228 IU/L to 170 IU/L at 6 months and to 34 IU/L at 12 months (reference range 23–46 IU/L). LDL cholesterol improved from 5.6 mmol/L to 3.6 mmol/L. Reported adverse effects included transient vomiting following dose escalation, which resolved spontaneously, and mild skin hyperpigmentation.

## Discussion

Acquired hypothalamic obesity remains one of the most challenging complications of hypothalamic injury. In the present report, we describe a child who developed severe early-onset obesity following neonatal *Escherichia coli* sepsis complicated by meningoencephalitis and brain abscess.

Acquired hypothalamic obesity results from the disruption of hypothalamic pathways that regulate appetite, satiety, and energy expenditure. Damage to hypothalamic nuclei leads to hyperphagia, reduced sympathetic tone, hyperinsulinemia, and a markedly decreased metabolic rate, resulting in rapid and often severe weight gain that is typically resistant to conventional lifestyle interventions (2, 7, 8). In addition to obesity, affected patients frequently develop pituitary hormone deficiencies, sleep and thermoregulatory disturbances, cognitive and behavioural impairment, and an increased risk of cardiometabolic complications (2).

Although hypothalamic obesity has classically been described in association with hypothalamic tumors, particularly craniopharyngioma and its treatment, the spectrum of acquired causes has expanded to include traumatic brain injury, inflammatory and infiltrative conditions, radiotherapy, and other structural brain insults (1, 2). Hypothalamic injury following neonatal meningoencephalitis remains extremely rare. Only isolated cases of hypothalamic obesity following infectious meningoencephalitis have been reported, highlighting the severity and rarity of hypothalamic injury in this context. To date, only a single case has been described in which hypothalamic obesity developed in a 7-year-old child after *Escherichia coli* meningoencephalitis.

The diagnosis of hypothalamic obesity in children is challenging due to its heterogeneous presentation, which varies according to age, aetiology, and the specific hypothalamic regions involved. Clinical features may evolve and are not always immediately suggestive of hypothalamic involvement (9). Radiological tools such as Puget’s classification are useful for estimating hypothalamic involvement in suprasellar tumours but are limited by subjectivity and modest predictive accuracy (10–12). Recent clinical algorithms emphasize the early identification of risk through careful monitoring of BMI trajectories and rapid weight gain, with timely referral for intervention to reduce long-term morbidity (13).

Until recently, treatment options for acquired hypothalamic obesity have been limited and largely ineffective. Multidisciplinary lifestyle programs may slow weight gain but rarely achieve meaningful or sustained weight loss, even when combined with pharmacologic agents such as metformin (14). Central stimulants, including dextroamphetamine and methylphenidate, have shown modest stabilization of BMI trajectories and improvements in hyperphagia and energy expenditure in selected patients, although their use requires careful monitoring for cardiovascular and neuropsychiatric adverse effects (15–17). Other approaches, such as intranasal oxytocin, have demonstrated inconsistent effects on weight despite acceptable tolerability (18). GLP-1 receptor agonists, including exenatide and semaglutide, have shown variable efficacy, with some patients achieving clinically meaningful weight loss while others show limited response (19, 20).

The emergence of melanocortin-4 receptor (MC4R) agonists has shifted the therapeutic landscape for acquired hypothalamic obesity. setmelanotide directly targets impaired melanocortin signaling, a core pathway disrupted in hypothalamic injury. Clinical studies have demonstrated significant reductions in BMI, hunger, and fat mass in both pediatric and adult patients with acquired hypothalamic obesity, with generally favourable safety profiles (4, 5). Most recently, an international Phase 3 randomized, placebo-controlled trial confirmed clinically meaningful BMI reduction and improvement in hyperphagia in patients aged 4 years and older, establishing setmelanotide as a targeted therapy for this otherwise refractory condition (5).

Recent real-world evidence from patients with Bardet–Biedl syndrome treated with setmelanotide has also highlighted potential benefits beyond weight reduction. Improvements in cognitive functioning and mobility have been described in case reports and patient-support program analyses (21, 22). In addition, treatment has been associated with improvements in metabolic dysfunction-associated steatotic liver disease (MASLD) and lipid parameters, with predominantly mild and transient gastrointestinal adverse effects (23). These observations are consistent with the improvements in motor activity, interaction, and metabolic parameters observed in our patient.

To our knowledge, this case report is the first to describe initiation of setmelanotide at 22 months of age in a child with acquired hypothalamic obesity following neonatal sepsis and meningoencephalitis. Treatment was associated with meaningful clinical benefit and was well tolerated, with mild skin hyperpigmentation as the only observed adverse effect, highlighting the importance of careful monitoring when using MC4R agonists in very young children.

While the findings from this single case are encouraging and align with improvements reported in older pediatric and adult cohorts, important limitations must be acknowledged. As an individual case report, conclusions regarding efficacy and long-term outcomes remain limited. Larger prospective studies will be necessary to define the safety, optimal dosing strategies, and long-term effects of early MC4R agonist therapy in children with acquired hypothalamic obesity, particularly inflammatory etiologies such as neonatal meningoencephalitis.

## Conclusion

This report supports the potential of early setmelanotide use in very young children with acquired HO, with manageable side effects and meaningful clinical benefits.

## Data Availability

The raw data supporting the conclusions of this article will be made available by the authors, without undue reservation.
