# Supplementary figures and images for: Case Report: Effect of setmelanotide treatment in a young patient with acquired hypothalamic obesity following Escherichia coli sepsis and meningoencephalitis with brain abscess
Source: Front Endocrinol (Lausanne). 2026 Jun 8;17:1796227. doi: 10.3389/fendo.2026.1796227 (PMC13283790; doi:10.3389/fendo.2026.1796227)

**Appendix1.**
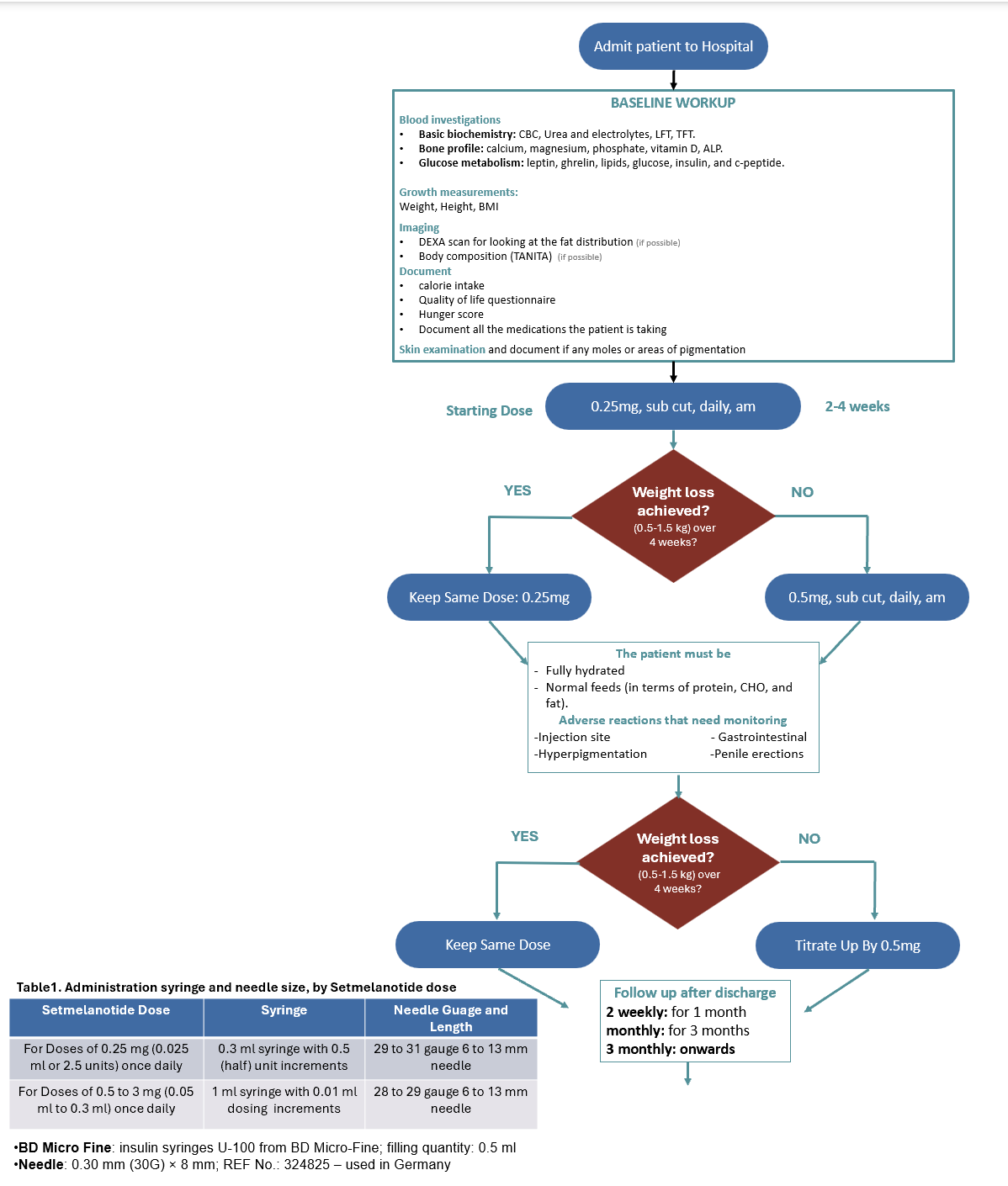
**PROTOCOL FOR STARTING SETMELANOTIDE IN A PATIENT WITH HYPOTHALAMIC OBESITY**
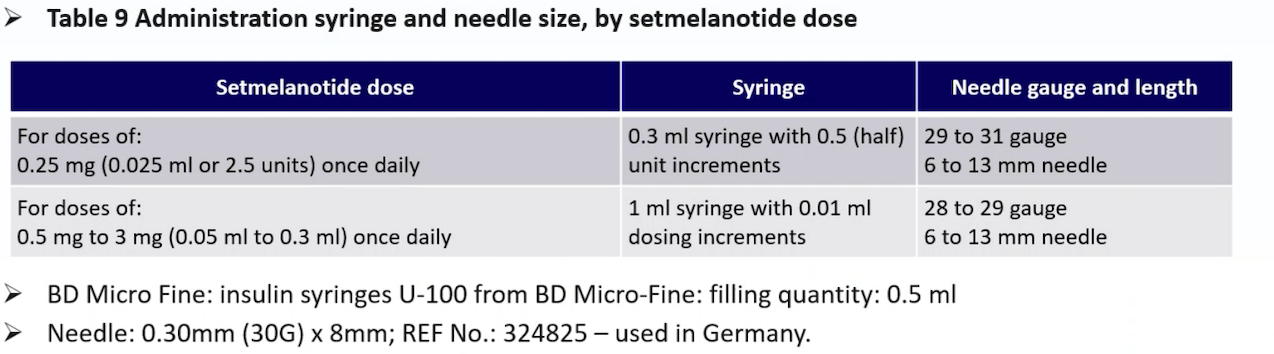

Supplement: Supplementary file 1 [file DataSheet1.docx]
